# Uptake of Workplace HIV Counselling and Testing: A Cluster-Randomised Trial in Zimbabwe

**DOI:** 10.1371/journal.pmed.0030238

**Published:** 2006-07-04

**Authors:** Elizabeth L Corbett, Ethel Dauya, Ronnie Matambo, Yin Bun Cheung, Beauty Makamure, Mary T Bassett, Steven Chandiwana, Shungu Munyati, Peter R Mason, Anthony E Butterworth, Peter Godfrey-Faussett, Richard J Hayes

**Affiliations:** **1**Department of Infectious and Tropical Diseases, London School of Hygiene and Tropical Medicine, London, United Kingdom; **2**Biomedical Research and Training Institute, Harare, Zimbabwe; **3**Department of Community Medicine, University of Zimbabwe, Harare, Zimbabwe; **4**Faculty of Health Sciences, University of the Witwatersrand, Witwatersrand, South Africa; **5**National Institute of Health Research, Harare, Zimbabwe; **6**Department of Medical and Laboratory Sciences, University of Zimbabwe, Harare, Zimbabwe; Harvard School of Public HealthUnited States of America

## Abstract

**Background:**

HIV counselling and testing is a key component of both HIV care and HIV prevention, but uptake is currently low. We investigated the impact of rapid HIV testing at the workplace on uptake of voluntary counselling and testing (VCT).

**Methods and Findings:**

The study was a cluster-randomised trial of two VCT strategies, with business occupational health clinics as the unit of randomisation. VCT was directly offered to all employees, followed by 2 y of open access to VCT and basic HIV care. Businesses were randomised to either on-site rapid HIV testing at their occupational clinic (11 businesses) or to vouchers for off-site VCT at a chain of free-standing centres also using rapid tests (11 businesses). Baseline anonymised HIV serology was requested from all employees.

HIV prevalence was 19.8% and 18.4%, respectively, at businesses randomised to on-site and off-site VCT. In total, 1,957 of 3,950 employees at clinics randomised to on-site testing had VCT (mean uptake by site 51.1%) compared to 586 of 3,532 employees taking vouchers at clinics randomised to off-site testing (mean uptake by site 19.2%). The risk ratio for on-site VCT compared to voucher uptake was 2.8 (95% confidence interval 1.8 to 3.8) after adjustment for potential confounders. Only 125 employees (mean uptake by site 4.3%) reported using their voucher, so that the true adjusted risk ratio for on-site compared to off-site VCT may have been as high as 12.5 (95% confidence interval 8.2 to 16.8).

**Conclusions:**

High-impact VCT strategies are urgently needed to maximise HIV prevention and access to care in Africa. VCT at the workplace offers the potential for high uptake when offered on-site and linked to basic HIV care. Convenience and accessibility appear to have critical roles in the acceptability of community-based VCT.

## Editors' Summary

**Background.:**

Since the first case of AIDS (acquired immunodeficiency syndrome) was reported 25 years ago, AIDS has become a major worldwide epidemic, with 3 million people dying from it in 2005. AIDS is caused by the human immunodeficiency virus (HIV), which is usually spread through unprotected sex with an infected partner. HIV damages the immune system, leaving infected individuals unable to fight off other viruses and bacteria. HIV infections can be treated with drugs know as “antiretrovirals,” and in an effort to deal with the global epidemic, world leaders have committed themselves to providing universal access to these drugs for everyone who needs them by 2010. Unfortunately, although access to antiretrovirals is rapidly increasing, so is the number of people infected with HIV. Last year, there were about 5 million new HIV infections, suggesting that more emphasis on prevention will be needed to halt or reverse the spread of HIV and AIDS. An important part of prevention is testing for HIV infection, but globally only 10% of people who need testing can access it. And even where such services are available, few people use them because of the stigma attached to HIV infection and fear of discrimination.

**Why Was This Study Done?:**

There is limited understanding about the factors that determine whether an individual will decide to have an HIV test. Yet, to reduce HIV spread, as many people at risk of infection must be tested as possible. Previous studies on VCT—a combination of voluntary testing and counseling about the implications of HIV infection and how to avoid transmitting the virus—have indicated that the convenience of getting the test, whether the test is directly offered, and the attitude of staff supplying it are all very important. In this study, the researchers asked whether providing VCT in the workplace could improve the “uptake” of HIV testing in Africa, where the HIV/AIDS epidemic is most widespread.

**What Did the Researchers Do and Find?:**

The researchers identified businesses with occupational health clinics in Zimbabwe, a country where 25% of adults carry HIV, and divided them into two “intervention” groups. Employees at half the businesses were offered “on-site VCT”—pre-test counseling followed by same-day on-site rapid testing, results, and post-test counseling. Employees at the other businesses had the same pre-test counseling but were offered a voucher for an HIV test at an off-site testing center and a later appointment to discuss the results—so-called off-site VCT. Everyone had the same access to limited HIV care should they need it. Although half of the employees at the on-site VCT businesses took up the option of HIV testing, only a fifth of employees at the off-site VCT businesses accepted vouchers for testing, and only one in five of these people actually used their voucher. This means that on-site VCT resulted in about 12 times as many HIV tests as off-site VCT. In both interventions, most of the people who accepted testing did so soon after entering the study and very few people were tested more than once. Finally, people 25 years old or younger, manual workers, and single people were most likely to accept testing in both interventions.

**What Do These Findings Mean?:**

These results suggest that on-site VCT in the workplace might be one way to improve uptake of HIV testing in Africa from its current low level and that providing VCT intermittently might be as effective as continuous provision. Importantly, say the researchers, the results of their study show that a relatively minor change in accessibility to testing can translate into a major difference in test uptake. This may hold true in non-occupational settings. However, these observations need to be repeated in more businesses and other settings, including those where there is no linked HIV care, before they can be generalized. Also, this study reports on the acceptability of this approach to providing VCT, but not on its impact on HIV prevention. As such the results do not indicate whether workplace VCT prevents HIV spread as effectively as other ways of delivering VCT. This will require research investigating how HIV incidence among HIV-negative employees and the partners of HIV-positive employees are affected by different VCT strategies.

**Additional Information.:**

Please access these Web sites via the online version of this summary at http://dx.doi.org/10.1371/journal.pmed.0030238.

• United States National Institute of Allergy and Infectious Diseases factsheet on HIV infection and AIDS

• United States Department of Health and Human Services information on HIV/AIDS treatment, prevention, and research

• US Centers for Disease Control and Prevention information on HIV/AIDS

• UNAIDS (Joint United Nations Programme on HIV/AIDS) information on political issues related to the HIV/AIDS epidemic and the 2004 UNAIDS/World Health Organization policy statement on HIV testing

• Aidsmap: information on HIV and AIDS provided by the charity NAM, which includes the latest scientific and political news

• MedlinePlus encyclopedia entry on HIV/AIDS

## Abstract 3

Voluntary counseling and testing for HIV has the potential for high uptake when it is offered on-site at the workplace and linked to basic HIV care.

## Introduction

Rapid scale-up of HIV care programs is ongoing in Africa, driven by ambitious targets set by the World Health Organization in 2003 and accompanied by the simultaneous need to intensify HIV prevention [ 1, 2]. HIV counselling and testing is a key component of both care and prevention, but has so far reached only a minority of Africans. A median of 9% of men and 7% of women reported ever having had an HIV test in surveys conducted in 25 African countries since 2000 [ 3].

Three previous randomised trials have compared uptake under different HIV testing strategies. These have established that rapid HIV testing with same day results reduced non-receipt of results, but had little effect on uptake when the indications for testing were pregnancy and sexually transmitted infections [ 4, 5]. For voluntary counselling and testing (VCT) in the community, however, uptake was significantly increased in Zambia by home-based delivery of results and counselling [ 6]. Observational studies have concurred that convenience, direct offer of testing, and positive attitude of staff have a critical impact on uptake of HIV testing, and appear to outweigh individual client-related factors [ 7– 13].

Workplace-based initiatives have the potential to expand access to HIV care in Africa at minimal cost to government [ 14]. We compared two VCT strategies linked to basic HIV care delivered through occupational clinics in small- and medium-sized businesses in Harare, Zimbabwe. A cluster-randomised design was used because the intervention was at the clinic level and the primary outcome concerned the potential benefit of providing VCT and HIV care at the workforce level. Zimbabwe has an estimated adult HIV prevalence of 25% [ 15]. Participating businesses were randomised either to counselling plus on-site rapid HIV testing or to counselling plus vouchers for off-site HIV testing at a chain of free-standing VCT centres. Both strategies were linked to the same package of basic HIV care. The aims were to compare the uptake of HIV testing and subsequent incidence of HIV-related morbidity and mortality under these two VCT/HIV care strategies. Here we report uptake.

## Methods

### Study Sites and Participants

Businesses operating within Harare were identified with the assistance of an HIV prevention project working with businesses (Zimbabwe AIDS Prevention Project), and were eligible if they had (i) 100 to 600 employees, (ii) an occupational or first aid clinic, and (iii) individual-based absenteeism records. Payrolls, used to identify all employees, were re-examined every three months for new employees and loss to employment. All employees expected to remain employed for at least 3 mo were eligible to participate.

### Randomisation

Businesses were categorised into high, medium, or low absenteeism strata using 3 mo of summary records. Allocation to the two VCT strategies used stratified randomisation within these three absenteeism strata (computer program written and run by ELC in S TATA 6.0; Stada, College Station, Texas, United States). Sample size was based on the potential impact of the intervention on health outcomes (to be reported separately). Randomisation occurred between enrolment and baseline interviews.

### Intervention

All eligible employees, including new employees during follow-up, were invited for interview when VCT was offered with pre-test counselling, individual risk assessment, and risk reduction plans. Participants at sites randomised to on-site rapid testing then had testing, results, and post-test counselling on the same day. Employees at sites randomised to the off-site strategy were given vouchers for off-site VCT and a 2-wk appointment to discuss results. The off-site VCT providers (New Start) had multiple branches in and around Harare situated at convenient locations (bus terminals and major shopping centres) that offered services after normal working hours and at weekends. After three reminders employees who had taken vouchers but reported not having used them were considered to have taken a voucher but not had VCT. This trial is reported in accordance with CONSORT guidelines ( Protocol S1). The study protocol, including the patient consent forms can be found in Protocol S2.

Counselling sessions were periodically observed by a supervisor who also conducted exit interviews. Debriefing meetings and refresher training were held every 2 wk and 6 mo, respectively.

HIV-positive employees were offered a package of care delivered through their occupational clinic, including post-test counselling, health education, vouchers for HIV testing of partners, and isoniazid preventive therapy and/or cotrimoxazole prophylaxis when indicated (tuberculin skin test positive with no evidence of active tuberculosis [TB] disease, and World Health Organization HIV clinical stages 2 to 4, respectively). The benefits of antiretroviral therapy, which was not part of this intervention, were discussed, and referral was made to local providers who became available during the second year of the study. Study nurses visited each clinic according to a schedule (at least three times a week) to provide ongoing HIV care, and open access to primary health care and the allocated VCT strategy for 2 y, after which service provision was continued by Zimbabwe AIDS Prevention and Support Organization. The start of intervention was staggered over 10 mo across the 22 businesses. Follow-up was for 2 y at each site and was completed for the last businesses in July 2004.

### Demography and Anonymised HIV Tests

All employees, whether accepting VCT or not, were asked to complete a questionnaire and provide venous or finger-prick blood or oral mucosal transudate for anonymised HIV testing, with written informed consent, at both the start and the finish of the intervention. Results of anonymised HIV tests were not made available to participants or clinic staff.

### Laboratory Methods

Anonymised HIV testing used Determine (Abbott, Wiesbaden, Germany) for blood and Vironostika II plus O (bioMerieux, Durham, North Carolina, United States) for oral mucosal transudate. One in ten specimens were retested for quality assurance (Unigold [Trinity Biotech, Dunblane, United Kingdom] for serum specimens, Vironostika II plus O for dried blood spots, and OraQuick [OraSure Technologies, Bethlehem, Pennsylvania, United States] for oral mucosal transudate).

On-site VCT was carried out by trained nurse-counsellors, using parallel Determine and Unigold with either venous or finger-prick blood. A written policy for discordant results and a quality assurance program were in place.

### Ethical Considerations

Anonymised HIV specimens were run, stored, and held apart from all personal identifiers other than a laboratory number. These were merged to other data using a linking file and computer program that immediately deleted all personal identifiers to maintain complete confidentiality during analysis.

Written informed consent was obtained from all participants. The study was approved by the ethics committees of the London School of Hygiene and Tropical Medicine and the Medical Research Council of Zimbabwe, Harare.

### Data Analysis

Comparison of the characteristics of participants used robust standard errors that took clustering at workplace level into account [ 16]. VCT uptake was analysed as a binary variable, because acceptance was not dependent on length of follow-up time, with most employees accepting shortly after direct offer: new employees were as likely to participate as those present for the entire study period. Only first episodes of VCT or voucher uptake were considered in the main analysis. Individuals moving between study businesses were considered under their first position only, so that any given individual could only account for one record. Associations between VCT uptake under each strategy and demographic and health variables were assessed by logistic regression, with robust standard errors. Based on cluster-randomised trial methodology, the data were reduced to 22 records [ 17, 18]. Wilcoxon rank sum tests were used to compare the proportions of participants who received VCT between the two arms of the trial. Risk ratios and 95% confidence intervals (CIs) are also reported.

Multivariate analysis was used to adjust for the stratified randomisation and other potential confounders, using logistic regression to predict the expected number of VCT users and the ratio of observed and expected numbers of events (standardised incidence ratio) as the outcome measure [ 17].

## Results

**Figure 1:**
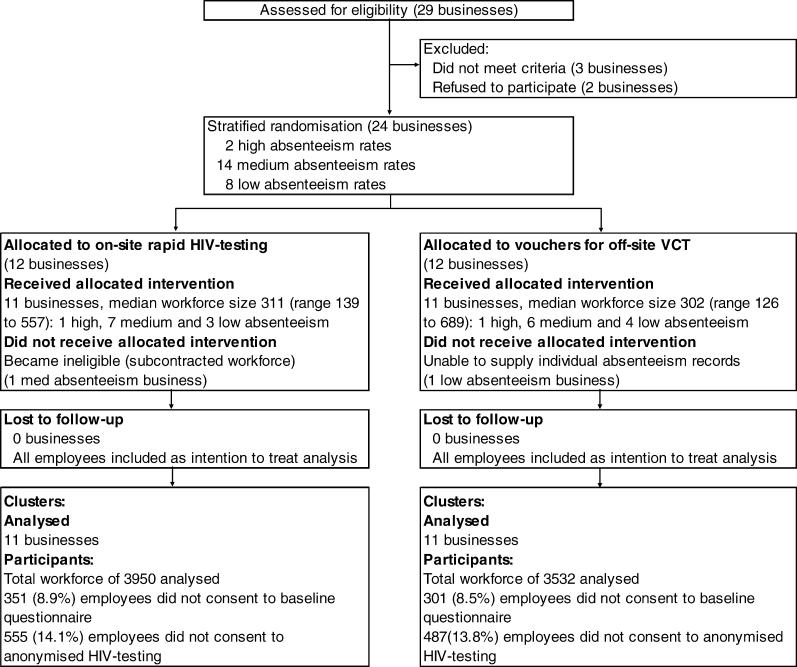
Trial Summary

A total of 29 businesses were assessed, of which 26 were eligible. Two withdrew before randomisation, and two were withdrawn between randomisation and intervention ( Figure 1). The cumulative number of employees during the 2-y intervention was 7,482 for the 22 participating businesses, including 1,305 employees who joined after the start of the intervention. Businesses were in manufacturing of hardware, construction, or industrial goods (14); clothing (3); food (3); and telecommunications (2). Participation rates were 91.3% for baseline interview and 86.0% for anonymised HIV testing. Baseline characteristics did not differ significantly by allocated VCT strategy, and are shown in Table 1.

**Table 1:**
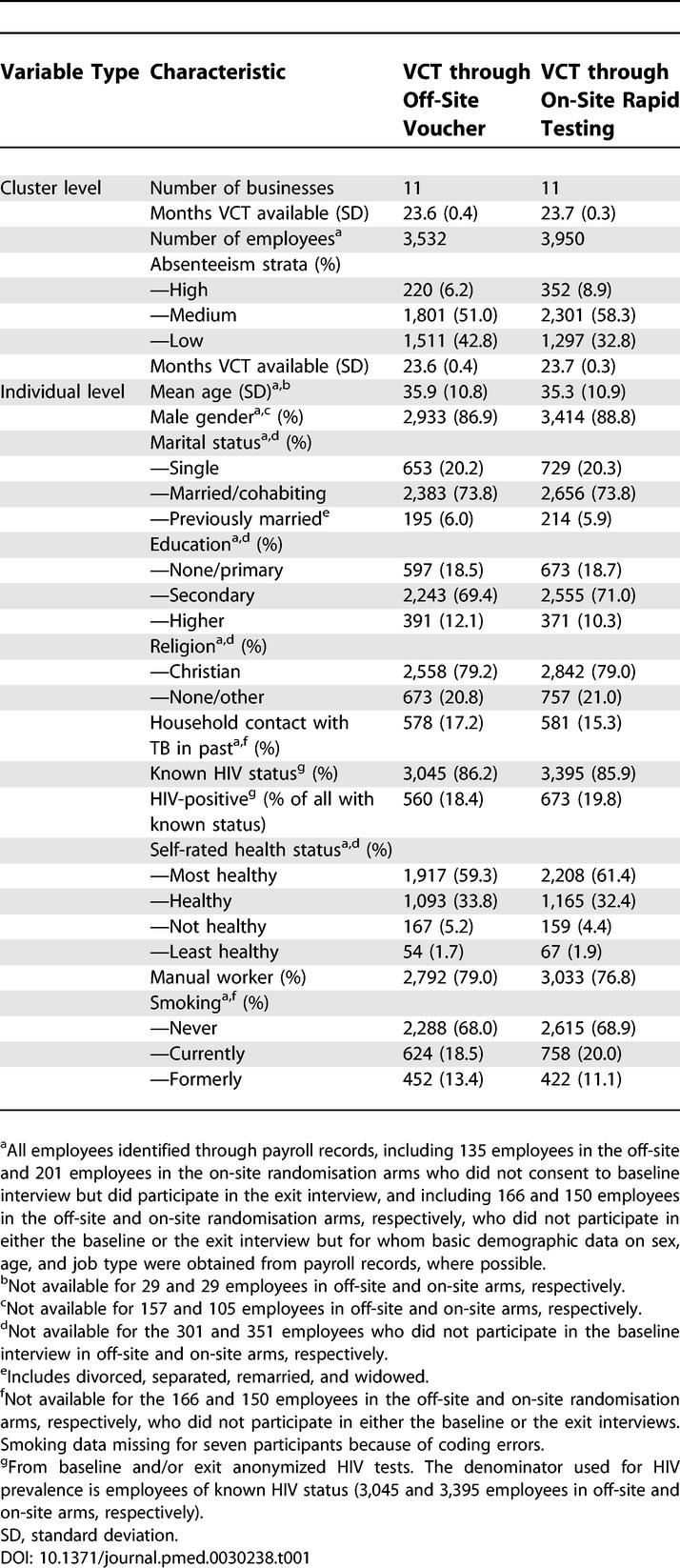
Baseline Characteristics according to Randomisation Group

### Uptake of On-Site HIV Testing Compared to Voucher Uptake

**Figure 2:**
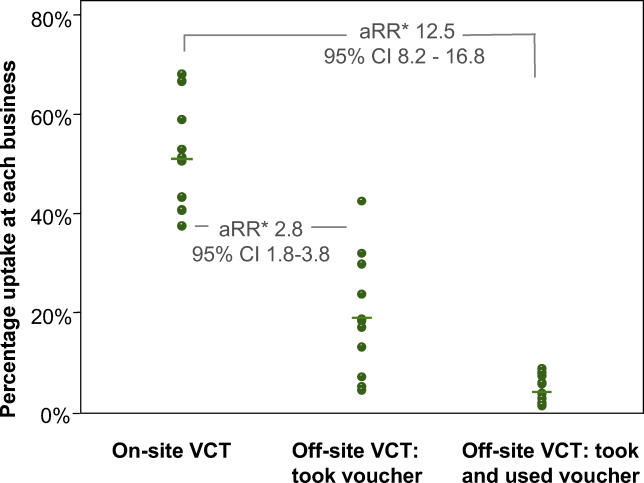
Uptake of VCT or Vouchers (Percentage) by Site and Allocated VCT Strategy Each point represents uptake at one business. The horizontal bars denote the mean site uptake within each category. aRR, risk ratio adjusted for age, sex, marital status, education, household contact with TB patients, self-rated health, and randomisation strata (see Table 2).

Uptake of VCT with on-site rapid testing was significantly and substantially greater than voucher uptake ( Figure 2; Table 2). A total of 1,957 employees at businesses allocated on-site rapid testing accepted VCT at least once (mean uptake by site 51.1%). There were no serious adverse events resulting from VCT. Under the off-site voucher strategy, 586 employees accepted vouchers at least once (mean uptake per site 19.2%). The difference was significant, with a risk ratio of 2.7 (95% CI 1.8 to 3.9) after adjustment for the cluster-randomised design ( Table 2). The risk ratio was 2.8 (95% CI 1.8 to 3.8) on further adjustment for differences in age, sex, marital status, education, self-rated health, and absenteeism strata.

**Table 2:**
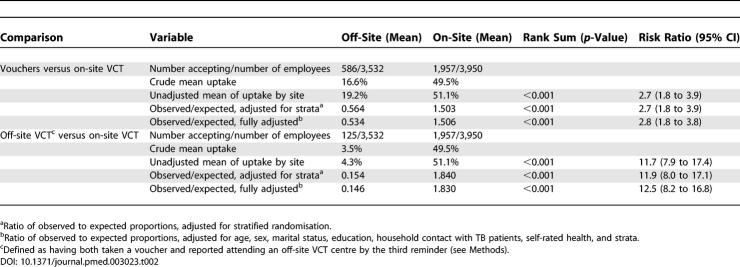
Uptake of Vouchers and Off-Site VCT versus Uptake of On-Site VCT

Previous VCT episodes were reported by 73 (12.5%) of the 586 employees who took vouchers and by 293 (15.0%) of employees accepting VCT at the workplace. A similar percentage of HIV-positive and HIV-negative clients reported previous VCT, suggesting that prior knowledge of HIV infection did not have a major impact on participation under either randomisation arm.

### Uptake of On-Site VCT Compared to Reported Off-Site VCT Attendance

Having accepted vouchers, only 125 employees reported having attended off-site VCT by their third reminder (21.3% of those taking vouchers; mean uptake per site 4.3%). Assuming that late or concealed attendance was negligible, the adjusted risk ratio for accepting on-site VCT compared to attending off-site VCT was 12.5 (95% CI 8.2 to 16.8), as shown in Table 2 and Figure 2.

### Time Course of First VCT Uptake

**Figure 3:**
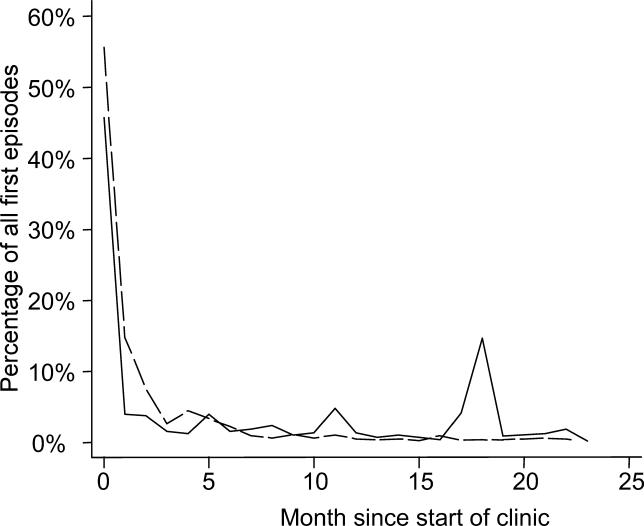
Timing of Uptake among Employees Who Accepted VCT or Vouchers Broken line denotes on-site VCT uptake; solid line denotes off-site voucher uptake.

VCT was available for 2-y at each site, but direct offer was made to each employee only once, unless HIV testing was indicated because of an HIV-related illness. The timing of first uptake among participants who accepted at least one on-site VCT or voucher is shown in Figure 3. Among employees who were present at the start of intervention, 1,239 (75.5%) of 1,640 who accepted VCT with rapid HIV testing did so within the first 2 mo of the intervention, with the corresponding proportion for voucher uptake being 274 (62.7%) of 437 participants. The high early uptake in part reflects the impact of direct offer, but there was obvious collective enthusiasm for on-site HIV testing, with demand outstripping counselling capacity in the first few weeks. Another possible manifestation of group dynamics, whereby employees encouraged one another to be tested, were the less prominent peaks in voucher uptake corresponding to periodic multiple requests for vouchers from a single site ( Figure 3).

### Multiple VCT Uptake

Individual employees were not limited to a single VCT episode, but could access services repeatedly for the 2-y study period, with the only constraint being a minimum time interval of 3 mo between VCT episodes. As part of post-test counselling, HIV-negative individuals were routinely encouraged to attend for repeat testing at 3 mo to exclude the “window period” of early HIV infection without detectable antibodies. However, apart from 388 participants in a sub-study investigating routine repeat testing at 3 mo [ 19], repeat episodes of VCT were uncommon, with only 9.6% of VCT clients in the on-site testing arm and 5.1% of clients in the off-site testing arm making repeat visits.

### Factors Associated with Accepting On-Site VCT or Vouchers for Off-Site VCT

Multivariate analysis of uptake of on-site VCT showed that the following factors were significantly associated with accepting VCT: age below 25 y (adjusted odds ratio [OR] 1.8; 95% CI 1.3 to 2.4) or 45 y or older (OR 1.7; 95% CI 1.1 to 2.8), being single (OR 1.3; 95% CI 1.1 to 1.5), having had past household exposure to TB (OR 1.2; 95% CI 1.0 to 1.4), having a manual job (OR 1.7; 95% CI 1.3 to 2.1), and poorer self-rated health (OR 1.4 per category below most healthy; 95% CI 1.2 to 1.7) ( Table 3). Although not significant in the univariate analysis, HIV status was significant in the multivariate analysis, with HIV-positive employees being less likely to test than their HIV-negative colleagues (OR 0.8; 95% CI 0.6 to 1.0).

**Table 3:**
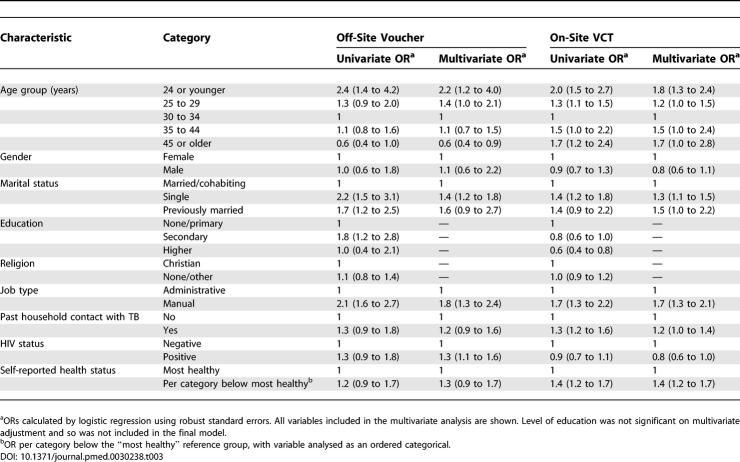
Univariate and Multivariate Adjusted OR (95% CI) of Factors Associated with VCT/Voucher Uptake

Table 3 also shows the multivariate analysis of voucher uptake in businesses allocated to off-site testing. Age below 25 y (OR 2.2; 95% CI 1.2 to 4.0), being single (OR 1.4; 95% CI 1.2 to 1.8), and having a manual job (OR 1.8; 95% CI 1.3 to 2.4) were significantly associated with accepting vouchers, while voucher uptake among those aged 45 y or older was significantly less likely (OR 0.6; 95% CI 0.4 to 0.9). As at businesses allocated rapid testing, HIV status was significant only in multivariate analysis, but in this case HIV-positive employees were more likely to take vouchers than HIV-negative employees (OR 1.3; 95% CI 1.1 to 1.6).

## Discussion

The results of this study demonstrate the high potential of rapid HIV testing and counselling at the workplace when this is linked to basic HIV care (not including antiretroviral therapy). Uptake of on-site rapid testing was significantly and substantially higher than that achieved through standard-of-care provision of free vouchers for off-site VCT linked to the same HIV care package (mean uptake of VCT by site 51.1% and 4.3%, respectively). The time course of uptake at our study sites ( Figure 3) suggests that periodic outreach VCT may be almost as effective as providing a continuous service. We have separately reported the high reproducibility of on-site HIV test results [ 19].

HIV testing and counselling is the gateway to HIV care, and may also contribute to HIV prevention by reducing high-risk sexual behaviour among individuals who know themselves to be HIV-positive [ 2, 20– 22]. VCT has been identified as one of the most cost-effective HIV/AIDS interventions in Africa [ 23]. Despite the high potential benefit, there is limited understanding of factors that determine acceptability and uptake, particularly for client-initiated testing outside of health-care settings.

Evidence of high readiness to test but limited means and motivation is available from other settings in Africa. Direct offer of HIV testing in a convenient location usually leads to high uptake in both health-care settings [ 7, 10, 24, 25] and community settings [ 6, 12, 13, 26]. Acceptance of provider-initiated testing can exceed 90% for antenatal clinic attendees and patients presenting with opportunistic infections [ 7, 10, 27], but with rates of return of only 45%–75% when a repeat visit is required [ 4, 27, 28]. However, only a minority of African adults will make unsolicited visits to free-standing or clinic-based VCT centres [ 29]. Major disincentives include fear of being seen, fear of breach of confidentiality, inability to cope or adverse life events if found to be positive, and a sense of futility if testing is not linked to HIV care [ 13, 30, 31]. Accessibility and cost are also important [ 13, 30, 31]. Making counselling and receipt of results available through home visits increased uptake of community-based VCT from 10%–12% to 37%–87% in four different African studies [ 6, 13, 26, 32]. Thus, the consistent finding is that relatively minor differences in accessibility translate into major differences in acceptability of VCT in Africa. Similar observations have been made in the United Kingdom and United States [ 5, 8].

Limitations of the current study are that the VCT intervention was combined with basic HIV care, so the acceptability of workplace VCT not linked to follow-up care is uncertain. Serious adverse reactions to testing HIV-positive, which we did not observe, could be more problematic when ongoing counselling is not provided. These factors may become less critical as public sector HIV care is scaled up in Africa. Businesses were identified through an organisation providing HIV prevention, and so there may be a selection bias towards those that are unusually receptive to addressing HIV issues. Operational studies of VCT uptake in the wider business community, with and without linked HIV care provision, are needed before our results can be more broadly generalised.

Individual factors associated with accepting VCT and vouchers were younger age, being single, manual work, suboptimal health, HIV status, and previous household contact with TB. The last may indicate greater readiness to test among individuals from HIV-affected households, as TB is strongly HIV-related in Harare [ 33]. Older employees were less likely to take vouchers but more likely to accept rapid VCT at the workplace than the reference age group of 30- to 34-y-olds, indicating that accessibility may be particularly important for this age group. In multivariate analysis that included self-rated health status, being HIV-positive significantly reduced the likelihood of accepting rapid VCT at the workplace but significantly increased the likelihood of accepting a voucher. Unadjusted HIV prevalence among clients accepting rapid VCT at the workplace was not significantly different from that of more comprehensive anonymised HIV testing, however, and the on-site strategy was still the most effective among HIV-infected persons.

A study of population-based counselling in Uganda also reported that HIV prevalence in VCT clients was very similar to that of anonymised HIV testing, but with over-representation of individuals with symptomatic HIV disease and under-representation of individuals with asymptomatic HIV infection [ 11, 12]. Taken with the current results, this suggests that high-impact VCT campaigns may provide useful population HIV prevalence estimates, but with over-estimation of the prevalence of symptomatic HIV and AIDS.

Policies towards the provision of diagnostic HIV testing services in Africa have undergone a major transformation in the last few years, from stressing the potential harm to stressing the need to achieve greater accessibility [ 34, 35]. Rapid HIV testing has minimal infrastructure requirements, can be performed accurately by non-laboratory personnel after brief training, and has expanded the potential scope of community-based VCT [ 35]. Further research is needed to confirm the high acceptability and to identify barriers to accepting rapid testing at the workplace. A key question will be whether VCT at the workplace, which essentially excludes couple counselling for most clients, is as effective for HIV prevention as other modes of delivering VCT. High-impact strategies aiming to “normalise” knowledge of HIV serostatus are under trial at present. Our results suggest that high accessibility will be a key requirement, and that including the workplace may contribute towards the realisation of this goal.

## Supporting Information

### Trial Registration

This trial has the registration number ISRCTN44114250 in the International Standard Randomized Controlled Trial Number Register.

Found at: http://www.controlled-trials.com/isrctn/trial/|/0/44114250.html.

Protocol S1 CONSORT Checklist (50 KB DOC)

Protocol S2 IPHC Workplace Protocol (459 KB PDF)

## Abbreviations

CI: confidence interval
OR: odds ratio
TB: tuberculosis
VCT: voluntary counselling and testing
