# Supplementary material for: Uptake of Workplace HIV Counselling and Testing: A Cluster-Randomised Trial in Zimbabwe
Source: PLoS Med. 2006 Jul 4;3(7):e238. doi: 10.1371/journal.pmed.0030238 (PMC1483908; doi:10.1371/journal.pmed.0030238)
Supplement: Supplementary file 1 [file pmed.0030238.sd002.pdf]

Intensified primary health care at the workplace:
Investigating the impact on HIV-associated morbidity and TB epidemiology.

Biomedical Research and Training Institute, Harare, Zimbabwe
Dr Liz Corbett, Professor Anthony Butterworth, Mrs Junior Mutsvangwa, Dr Steven Chandiwana, Professor Peter Mason

Blair Research Institute, Harare, Zimbabwe
Mrs Shungu Munyati

Rockefeller Foundation
Professor Mary Bassett

London School of Hygiene and Tropical Medicine, Keppel Street, London, UK
Dr Peter Godfrey-Faussett, Dr Maria Quigley, Prof. Richard Hayes

University of Oxford
Dr Ajit Lalvani

TABLE OF CONTENTS

TABLE OF CONTENTS 2

TABLE OF APPENDICIES 3

1 SUMMARY 4

1.1 BACKGROUND 4
1.2 SETTING 4
1.3 OBJECTIVES 4
1.4 DESIGN 4
1.5 STUDY POPULATION AND SITES 4
1.6 STUDY DURATION 4
1.7 PRIMARY OUTCOMES 4
1.8 SECONDARY OUTCOMES 5
1.9 STRATIFICATION AND FOLLOW-UP 5
1.10 ADDITIONAL STUDIES TO BE CONDUCTED UNDER THE SAME STUDY FRAME-WORK 5

2 OBJECTIVES 5

2.1 SETTING 6
2.2 DESIGN 6

3 BASELINE SURVEY, SURVEILLANCE SPECIMENS AND ONGOING DEMOGRAPHY 6

3.1 ABSENTEEISM RECORDS 6
3.2 DEFINING THE BASELINE COHORT 6
3.3 FOLLOWING SUBSEQUENT EVENTS 6
3.3.1 Baseline one-to-one interviews *7*
3.3.2 Baseline biological specimens *7*

4 SERVICES PROVIDED AT ALL ON- AND OFF-SITE CLINICS 7

4.1 HIV TESTING 7
4.1.1 On-site VCT *7*
4.1.2 Off-site VCT *7*
4.2 HIV-TESTING PROMPTED BY ILL HEALTH 7
4.3 PREVENTIVE THERAPY (SEE ALSO SECTION 6 FOR MORE DETAIL) 8
4.4 SERVICES PROVIDED FOR SPOUSES AND PARTNERS OF PARTICIPATING EMPLOYEES 8
4.4.1 HIV-testing *8*
4.4.2 Preventive therapy *8*
4.4.3 STD management *8*
4.4.4 TB contact tracing *8*
4.5 DIFFERENCES BETWEEN SITES WITH NEWLY ESTABLISHED CLINICS AND THOSE WITH PRE-EXISTING CLINICS 9
4.5.1 Newly established clinics *9*
4.5.2 Pre-existing clinics *9*
4.5.3 Tasks that will remain the sole responsibility of research staff *9*

5 INITIATION AND FOLLOW-UP OF PATIENTS TAKING PREVENTIVE THERAPY 9

5.1 ISONIAZID 9
5.1.1 Duration and dose of therapy *9*
5.1.2 Exclusion criteria *9*
5.1.3 Inclusion criteria *10*
5.1.4 Screening for eligibility *10*
5.1.5 Starting and following-up clients on isoniazid *10*
5.1.6 Prevention and management of side-effects *11*
5.2 COTRIMOXAZOLE 11

5.2.1 Duration and dose of therapy ... *11*
5.2.2 Exclusion criteria ... *11*
5.2.3 Inclusion criteria ... *11*
5.2.4 Screening for eligibility ... *11*
5.2.5 Starting and following-up clients on cotrimoxazole ... *11*
5.2.6 Prevention and management of side-effects ... *11*
5.3 MEASUREMENT OF ADHERENCE TO IPT AND COTRIMOXAZOLE ... 12

6 TB DIAGNOSIS, CASE-DEFINITIONS AND MANAGEMENT ... 12

6.1 INVESTIGATION OF TB SUSPECTS ... 12
6.2 CONFIRMED TB ... 12
6.3 TB TREATMENT REGIMENS ... 12

7 WORKFORCE TURNOVER ... 13

7.1 NEW EMPLOYEES ... 13
7.2 EMPLOYEES LEAVING THE STUDY WORKFORCE ... 13

8 OUTCOME MEASURES ... 13

8.1 PRIMARY OUTCOMES ... 13
8.2 SECONDARY OUTCOMES ... 13
8.3 CASE DEFINITIONS ... 14
8.3.1 TB ... *14*
8.3.2 Severe morbidity ... *14*
8.4 ANALYSIS ... 14

9 LABORATORY METHODS ... 15

9.1 SURVEILLANCE SPECIMENS ... 15
9.1.1 Processing and storage of baseline blood specimens ... *15*
9.1.2 Ongoing routine 2 monthly sputum smears ... *15*
9.2 DIAGNOSTIC HIV TESTS ... 16
9.2.1 VCT in the industrial sites ... *16*
9.2.2 Routine quality assurance ... *16*
9.2.3 Laboratory diagnostic HIV tests ... *16*
9.3 DIAGNOSTIC TB SMEAR AND CULTURE METHODS ... 17
9.3.1 Diagnostic TB specimens ... *17*
9.3.2 Diagnostic TB cultures ... *17*

10 TREATMENT INTERRUPTION ... 17

TABLE OF APPENDICIES

APPENDIX 1. MANAGEMENT OF ADVERSE EVENTS, DRUG INTERACTIONS AND CONTRAINDICATIONS TO ISONIAZID AND COTRIMOXAZOLE ... 18

1 Summary

1.1 Background

Meeting increasing health care needs without compromising quality and standards of care is a major challenge facing countries that have been severely affected by the HIV epidemic. In areas of limited resources, the development and implementation of guidelines for integrated care practice that cover common adult conditions offers the potential to improve quality and outcome at low cost, while minimising waste of resources from inappropriate prescribing and referral habits. Diagnosis and management of HIV-associated conditions are among the key areas that need to be strengthened at the primary health care level.

HIV is a strong risk factor for TB, increasing both the risk of rapidly progressive TB disease following TB infection, and the rate of reactivation in those with latent TB infection. Because of this interaction, TB disease is one of the most common HIV-associated opportunistic infection in countries where latent TB infection is widespread. In some countries in sub-Saharan Africa, TB incidence rates have increased up to 4 fold. It is not yet clear, however, what impact this has had on TB transmission rates, or how control can best be improved in high HIV prevalence countries.

1.2 Setting

20 to 24 industrial health clinics in Harare, Zimbabwe, collectively serving about 6,000 employees. Industrial clinics differ from public primary health clinics in several important respects. They also have the advantages of being run by one or two multipurpose general nurses and serving a discrete population. Both factors facilitate epidemiological investigation and follow-up.

An important additional advantage is that interventions aimed at preventing illnesses have a high potential to be cost-effective in the industrial setting, and therefore sustainable, because of the considerable direct and indirect costs incurred by the employer in the event of ill-health among their employees.

1.3 Objectives

- to investigate the acceptability and feasibility of promoting voluntary counselling and HIV-testing (VCT) at the workplace
- to investigate the impact of intensified primary health care, including provision of VCT and preventive therapy for persons found to be HIV+ve, on HIV-associated morbidity and TB control at a population level

1.4 Design

Open cluster-randomised cohort study comparing off-site versus on-site VCT, linked to a package of on-site intensified primary health care

1.5 Study population and sites

The study population will be the employees of 20 to 24 participating companies

1.6 Study duration

The total study duration is 4 years. The preparation, recruitment, follow up, data analysis and write up will occur over 9, 6, 24 and 3 months periods respectively, with the recruitment and follow-up periods over 6 months between the different companies

1.7 Primary outcomes

a) The difference in i) absenteeism rate due to sickness (clinic visits, sick leave, hospitalisation) and ii) incidence of serious morbidity events (see definitions below) between the two arms.
b) The acceptability of VCT and proportion of HIV-positive employees in each arm identified and screened for preventive therapy eligibility

1.8 Secondary outcomes

The difference between the two arms in:-

a) absenteeism rates among all HIV-positive employees
b) incidence of serious morbidity events (hospitalisation, death or medical retirement) among all HIV-positive employees
c) incidence of TB during the last 12 months of the intervention
d) prevalence of active TB detected on a cross-sectional survey at the end of the intervention at each site

1.9 Stratification and follow-up

20 - 24 companies will be grouped into 4 strata according to size and pre-intervention absenteeism rates. Companies will then be randomly allocated to either on-site or off-site VCT services and will then be followed-up for two years, aiming for a combined workforce of about 3000 in each arm.

1.10 Additional studies to be conducted under the same study frame-work

a) Studies investigating the natural history of HIV infection at the primary health care level, including:-
   - the impact of HIV infection on consultation frequency, case mix and outcome of common pre-defined presenting syndromes
   - the impact of HIV infection on aspects of health economics, including absenteeism rates and indices of productivity
   - the interaction between smoking and HIV infection on respiratory health (with Mrs Shungu Munyati)

b) A household contact study to investigate TB transmission rates from factory workers to their household. The study with investigate
   - the prevalence of TB infection between household contacts of workers with TB and those without TB, and
   - household contacts of workers with HIV-associated TB and HIV-negative TB
   - the association between infectivity and duration of smear positivity before diagnosis

   All TB patients identified during the factory-clinic study, together with a systematic selection of control subjects, will be invited to participate. Duration of smear positivity prior to treatment will be determined from a library of unstained sputum smears to be collected and stored routinely from all 6000 workers every 2 months. TB infection will be identified both by tuberculin skin testing (TST), and by enumeration of *M. tuberculosis* specific T-lymphocyte cells using the *ex-vivo* ELISPOT technique

c) A cross-sectional study of TB prevalence. At the end of the factory intervention study 3 sputum smears and cultures will be taken from all workers. The impact (if any) of HIV infection on duration of smear positivity, and on prevalence of active TB and chronic TB (defined as > 6 mos of smear positivity before diagnosis) will be investigated.

d) A health economics study to investigate the cost-effectiveness to businesses of providing the on-site VCT service and intensified health care intervention for their employees.

2 Objectives

To investigate whether providing on-site voluntary counselling and testing (VCT) and preventive therapy (isoniazid and cotrimoxazole for persons found to be HIV-positive), is acceptable and able to reduce absenteeism due to ill-health, prevalent and incident TB and severe morbidity at the work force level. If so, the cost-effectiveness for businesses of providing these services will be investigated.

Comparison will be made with sites provided with the same preventive therapy services that are linked to HIV diagnosis through an off-site VCT centre.

2.1 Setting

Medium sized businesses located in the light industrial area of Harare, mainly selected from companies that have already participated in a primary HIV prevention project run by a collaborating group (ZAPP). Choice of company is based on:

- convenience of location
- size (not less than 100 and not more than permanent 500 employees)
- workforce stability (minimal seasonal fluctuations or reliance on short-term employees)
- willing to accommodate research needs and provide the baseline data required for the initial project assessment.

The minimum and minimum sizes of companies were set as 100 and 500 employees respectively in order to maximise power and enable all sites to be covered with the existing staff quota.

2.2 Design

An open cluster-randomised cohort study. Clusters will be comprised of employees at individual companies.

Companies will be stratified into 4 groups according to size and pre-intervention absenteeism rates. Within each strata, sites will be randomisation to receive either on-site or off-site VCT services.

3 Baseline survey, surveillance specimens and ongoing demography

3.1 Absenteeism records

Records of workforce size, turnover, and absenteeism due to ill-health will be collected each week (or month for factories unable to supply weekly records) onto a standardised proforma. Data covering a 3 month period will be collected and analysed in order to stratify companies prior to randomisation.

3.2 Defining the baseline cohort

A list of all employees giving first name, surname, company number, and if possible, date of birth, will be requested from each company immediately prior to, or at, the initiation of services at each site.

For each company, a database table defining the starting workforce (referred to as cohort from herein) will be established from the pay records.

3.3 Following subsequent events

Once the initial cohort has been defined, workforce turnover at each site will be monitored weekly (or monthly for companies unable to supply weekly data). Personnel or payroll officers will be asked to complete this form each week/month, providing the project with notification of all newly recruited staff, and the date of leaving employment and reason why will be recorded for all those lost to employment during the study period.

At each site the cohort will be kept up-to-date by weekly/monthly monitoring and data entry of any new employee details, including date of recruitment. Employees who leave employment will remain in the analysis, but the date at which they leave, and reason for leaving will be recorded for use in all subsequent cohort analyses.

3.3.1 Baseline one-to-one interviews
On initiation of services at each site a one-to-one interview will be requested with each employee. Information will be collected using a standardised questionnaire (BD01). At this time all employees will be asked to provide venous blood and sputum specimens, with written consent.

A patient information leaflet will be given to all employees who attend the on-to-one interview, explaining the purpose of the intervention and nature of the biological specimens requested.

It will be made clear that participation in the on-to-one is entirely voluntary, as is the giving of biological specimens.

3.3.2 Baseline biological specimens
A sample of venous blood and sputum will be requested from each employee at the one-to-one interview. A dried blood spot made from a finger prick blood sample will be requested from each participant who is unwilling to give a venous blood sample.

For laboratory methods see section 9.

4 Services provided at all on- and off-site clinics

Routine clinic services will be available at all sites, regardless of HIV status and regardless of the extent to which any individual employee has participated in other aspects of the project.

As far as possible, a standardised syndromic approach will be used for management of common complaints, as defined by the "Integrated management of adult illnesses" (see IMAI manual, desk aide and data capture form IM01).

4.1 HIV testing

4.1.1 On-site VCT
VCT will be available for all employees who wish to know their HIV status. VCT will be available as on-site brief pre-test counselling, followed by rapid testing with written consent and results available one hour after testing. Participants do not have to know their results, even if they have had a test carried out. Brief post-test counselling will be provided by the clinic nurses, who will also be available for on-going psychological support as needed.

4.1.2 Off-site VCT
For the off-site centres, brief on-site pre-test counselling will be provided followed by provision of pre-paid vouchers for "New Start Centre" free-standing VCT sites. Clients will not be provided with transport or time off work to visit the VCT centre.

An appointment will be made for 2 weeks after the voucher has been given, which the client does not have to keep, to give the client the opportunity to share the VCT results with the clinic nurse if desired. This will be necessary for any HIV-positive employee wishing to commence preventive therapy.

If a client discloses that they are HIV-positive then a blood specimen will be drawn and sent to the study laboratory for repeat testing, because written confirmation is not provided by New Start Centres.

4.2 HIV-testing prompted by ill health
In addition to providing access to VCT, the project will promote and streamline HIV-testing for health-related indications. A number of conditions are defined as being HIV-associated in the syndromic management guidelines.

These are conditions that either occur with increased frequency in the context of immunosuppression (such as oral herpes, pneumonia, diarrhoea, and TB) or are associated with increased risk of HIV acquision, such as STIs. Some conditions, notably genital herpes, fall into both risk categories.

At all sites using the syndromic management approach, the diagnosis of such a condition will prompt discussion of the association with HIV infection, and advise to have an HIV-test. For clients who accept the offer of HIV-testing, the test will be carried out as for VCT above (i.e. will differ between sites). In all cases:

- written consent will be obtained before on-site testing
- the indication, offer and acceptance of VCT, or a voucher, will be recorded on the IMO1 form for that illness episode
- in the case of off-site testing, an appointment will be made for 2 weeks to discuss the HIV-test results. If the patient still has not visited New Start Centre, but wishes to know their HIV status, then a blood specimen may be sent to the laboratory for off-site HIV testing and a follow-up appointment made for 2 weeks time
- persons reporting a positive test result at New Start Centre will be re-tested as part of their screening for preventive therapy eligibility to confirm the result

4.3 Preventive therapy (see also section 6 for more detail)

Persons found to be HIV-positive will be screened for eligibility for preventive therapy with isoniazid (if tuberculin skin test positive; active TB excluded; no TB treatment for the last 2 years and no contra-indications) and/or cotrimoxazole (WHO stage 2, 3 or 4 and no contraindications) using the PT01 assessment form.

Patients started on isoniazid will be monitored monthly for side-effects and compliance. A booklet, IH01, will be started for each patient starting isoniazid, and will be sent in to BRTI once follow-up is complete. IH01 monitors side-effects, which should be reported immediately using PT04, and contains instructions as to when to prolong therapy based on the pill count for each patient.

All known HIV-positive patients will be followed-up 3 monthly and reassessed for WHO stage. Cotrimoxazole eligibility will be reassessed 3 monthly for patients not already on contrimoxazole. Progress and therapy will be monitored and reported on a 3 monthly basis using forms PT02 and PT03 respectively. Adverse events will be reported without delay using PT04.

4.4 Services provided for spouses and partners of participating employees

4.4.1 HIV-testing

Pre-paid New Start Centre Vouchers will be provided for spouses and sexual partners of VCT participants

4.4.2 Preventive therapy

The study remit does not extend to providing isoniazid or cotrimoxazole to spouses or partners of project participants. An information leaflet will be provided to inform participants about the dangers of "sharing" medicines, and to encourage their partners to discuss their HIV status and preventive therapy with their own health care provider.

4.4.3 STD management

Partner notification slips will be provided for employees diagnosed as having an STI, and the partner will be encouraged to seek treatment from their local clinic

4.4.4 TB contact tracing

Any participant diagnosed as having TB during the course of the study will be asked for permission to conduct a household contact screening of their household, as detailed in protocols

PHH01 and PHH02. This will only apply to participants with *active TB disease*, and not with *inactive latent TB infection*

4.5 Differences between sites with newly established clinics and those with pre-existing clinics

4.5.1 Newly established clinics

The IMAI syndromic management approach will be used in all newly established project clinics, which will staffed by trained project nurses on a part-time basis. Once established, a nurse will attend the clinic on a fixed schedule of between 2 and 5 half days per week, depending on the workforce size.

4.5.2 Pre-existing clinics

The project aims to integrate with on-going services at pre-existing sites, rather than replacing them or acting in parallel. Training of company-employed nurses in the use of IMAI will be provided, as will the requisite IMAI manual and desk-aide.

Non-project employed nurses will be encouraged to change their practice to the IMAI approach following training, and to participate in all project activities following initial supervision from senior research nurses. As and when both company and project nurses are confident that standard procedures are being followed, shared tasks to be carried out by the company nurse can include:

- syndromic management using the IMAI approach
- ongoing use and filling in of the IMAI data capture sheets (IM01)
- issuing advise to have an HIV test when indicated by the IMAI
- completing the VCT risk analysis and delivery of pre-test information and counselling
- issuing VCT vouchers at the off-site clinics
- following-up VCT clients with their results at the two week post-test assessment
- booking in clients to receive VCT from project nurse at the on-site clinics
- conducting follow-up visits after a positive test, including carrying out the WHO staging
- following-up patients on cotrimoxazole and isoniazid preventive therapy.

4.5.3 Tasks that will remain the sole responsibility of research staff

The following will *always* be carried out by project staff, and should *not* be delegated to non-project nursing staff, however willing and competent.

- Running rapid HIV tests on-site and giving patients their results
- Placing and reading tuberculin skin tests
- **Initiating** isoniazid and cotrimoxazole preventive therapy

Each site will be visited by project staff, normally the supervising senior research nurse, at regular pre-arranged times to provide VCT, and preventive therapy screening, drugs and follow-up as an ongoing service. In order to maintain confidentiality, project staff will attend each site with non-project staff not less than two half-days per week, and will perform a wide range of duties, including syndromic management of unselected clients, as well as carrying out VCT and follow-ups.

5 Initiation and follow-up of patients taking preventive therapy

5.1 Isoniazid

5.1.1 Duration and dose of therapy

300mg of isoniazid daily for 6 months

5.1.2 Exclusion criteria

- HIV-negative or status unknown
- Active TB confirmed, or cannot be excluded
- TB disease diagnosed within the last 2 years
- Known isoniazid intolerance

- Known cirrhosis or chronic liver disease

5.1.3 Inclusion criteria

- HIV-positive *plus*
  - Tuberculin skin test (TST) positive (≥ 5mm induration)
  - OR TB disease more than 2 years ago
- ➢ **If previously diagnosed with TB disease, then the TST is not informative and should be omitted. In that case, offer isoniazid if otherwise eligible *and* last TB treatment was more than 2 yrs ago**

5.1.4 Screening for eligibility

- Once HIV infection is diagnosed, eligibility for preventive therapy will be assessed from the questionnaire PT01 and the continued care record CC01F.

5.1.4.1 Excluding active TB

- Persons with positive TST results (≥ 5 mm induration) who have no symptoms of active TB will be started on isoniazid without further screening
- Persons with one or more symptoms compatible with active TB will be worked up as a TB suspect with:
  - ➢ 3 sputum smears
  - ➢ amoxycillin (or ampicillin) 500 mg tds for 7 days
  - ➢ results and response will be reviewed in 5 - 7 days to assess: smear positive? completely better on antibiotics?
    - ❖ *either* confirm normal chest radiography before starting isoniazid if smear negative and symptoms have completely resolved on antibiotics
    - ❖ *or* referral as smear positive TB if one or more smears are positive
  - ➢ If smear negative, but still symptomatic then 3 further sputum smears will be taken and the patient given a second antibiotic (erythromycin 500 mg qds)
    - ❖ the client will be referred as a smear negative TB suspect if still smear negative and symptomatic at the end of the second course of antibiotics, and isoniazid will not be started

5.1.4.2 Excluding clients for other reasons

- PT01 includes questions aimed at detecting clients who have had previous adverse reactions to TB treatment or isoniazid, or who have known chronic liver disease

5.1.5 Starting and following-up clients on isoniazid

- An IH01 form will be started for all patients starting isoniazid and the CCF01 form will be completed to maintain the clinic records
- Isoniazid will be given as 300mg tablets, one tablet to be taken daily for 6 months
  - ➢ Isoniazid therapy will be self-administered
  - ➢ Clients will be warned to discontinue their drugs and report symptoms suggestive of isoniazid toxicity promptly
  - ➢ A starter pack of 40 tablets will be supplied at the initial visit
  - ➢ 40 tablets of pyridoxine 50mg will also be given to clients who have symptoms of peripheral neuropathy
  - ➢ A follow-up appointment will be made for 1 month
  - ➢ Clients will be asked to bring back their medication packet, with any remaining tablets to the follow-up appointment
- Patients will be reviewed monthly while on treatment
  - ➢ The relevant section of IH01 will be completed at each visit
  - ➢ Management of adverse events will be as indicated in IH01, but should be reported immediately using PT04
  - ➢ Every 3 months a PT03 reporting form will be filled in and submitted
  - ➢ After 6 months the compliance of the client will be reassessed, and the pill count summary section of IH01 used to assess the need for further isoniazid therapy.
  - ➢ Once treatment is complete (or has been discontinued), the IH01 form will be submitted to BRTI

5.1.6 Prevention and management of side-effects
Only one month's supply of isoniazid will be dispensed at each visit. Patients receiving IPT will be warned to discontinue the drug immediately and report symptoms suggestive of hepatitis, such as nausea, vomiting, dark urine or jaundice. Patients will be monitored clinically once a month at the primary health care clinic while on IPT, with monitoring of liver function tests if symptoms are reported.

Pyridoxine supplementation will not be routinely provided. However, pyridoxine will be provided to all participants who develop peripheral neuropathy.

5.2 Cotrimoxazole

5.2.1 Duration and dose of therapy
960 mg of cotrimoxazole daily, to be continued indefinitely

5.2.2 Exclusion criteria
- HIV-negative or status unknown
- WHO stage 1 HIV disease
- Allergic to cotrimoxazole

5.2.3 Inclusion criteria
- HIV-positive *plus*
  - WHO stage 2, 3 or 4 disease

5.2.4 Screening for eligibility
- Once HIV infection is diagnosed, eligibility will be assessed from the questionnaire PT01 and continued care record CC01F
- All HIV-positive clients who are WHO stage 2, 3 or 4 disease and have no known contra-indication will be offered cotrimoxazole

5.2.5 Starting and following-up clients on cotrimoxazole
- Initial follow-up for patients starting cotrimoxazole will be using CC01F for the 1st month, and then PT02 from 1 month on.
  - A PT03 progress form will be filled in 3 monthly for all patients on cotrimoxazole and sent into BRTI
  - Adverse events will be reported immediately using PT04
- Cotrimoxazole will be given as 480 mg tablets, with two tablets taken each day
  - ➢ Cotrimoxazole will be self-administered
  - ➢ Clients will be warned to discontinue their drugs and report rashes or burning sensations in the skin promptly
  - ➢ A starter pack of 40 tablets will be supplied at the initial visit
  - ➢ A follow-up appointment will be made for 1 month
    - ❖ clients will be asked to bring their medication packet, with any remaining tablets to the follow-up appointment
    - ❖ a full blood count, including haemoglobin, total white cell count and platelet count, will be taken, and the client reviewed with the results in 1 weeks time
  - ➢ Management of adverse events will be as indicated in PT02 and reported using PT04
- Clients with no adverse events will be reviewed 3 monthly while on treatment, and will come once monthly to pick up packets of 30 tablets
  - ➢ A PT03 form will be completed at each visit, and relevant PT02 section filled in to identify break-though TB disease, non-compliance with treatment, and treatment side-effects.

5.2.6 Prevention and management of side-effects
Only one month's supply of cotrimoxazole will be dispensed at each visit, and patients will be warned to discontinue the drug and report to the clinic immediately if they develop a rash

A full blood count will be taken at the end of 1 months therapy. Cotrimoxazole will be stopped and folic acid started if:-
- *wbc < 1.5* ***OR***

- *neutrophil count < 1.0 OR*
- *Plts < 100*
- *Hb < 8.0.*

Otherwise the blood count will be repeated in 1 month if lesser abnormalities are present, or 6 monthly if normal.

Symptoms of nausea/vomiting will be managed by taking the drug at night time, or reducing the dosing interval to alternate days

All suspected adverse events will be reported using PT04

5.3 Measurement of adherence to IPT and cotrimoxazole

Adherence will be assessed using;

- The proportion of monthly treatment collected
- The number of pills returned each month

6 TB diagnosis, case-definitions and management

6.1 Investigation of TB suspects

The indications for suspecting TB will be as defined in the IMAI

- TB suspects will have 3 sputum smears taken and be started on a 7 day course of amoxycillin 500 mg tds (erythromycin 500 mg qds if penicillin allergic)
- VCT will be offered at the first visit if HIV status is unknown or previously negative
- Results and response to treatment will be reviewed in 5 to 7 days
  - Smear-positive?
    - refer as *confirmed smear positive TB*
  - smear negative and completely better on antibiotics?
    - classify illness as pneumonia and discharge
    - if a candidate for isoniazid, then a chest radiography will be taken before starting isoniazid
  - Smear-negative and still symptomatic despite antibiotics?
    - repeat 3 sputum smears and give a second course of antibiotics for 1 week (erythromycin 500 mg qds, or doxycycline 200mg bd)
    - Results and response to treatment will be reviewed in 5 to 7 days
      - smear positive?
        - refer as *confirmed smear positive TB*
      - smear negative and completely better on antibiotics?
        - classify illness as pneumonia and discharge
        - if a candidate for isoniazid, then a chest radiography will be taken before starting isoniazid
      - smear negative and still symptomatic despite antibiotics?
        - refer as *smear negative TB suspect*

6.2 Confirmed TB

Patients with confirmed smear positive or culture positive TB will be referred to the City Health Clinics for further management, and will be offered the option of workplace DOTS.

- These patients will be reviewed by the Household Contact Study team

6.3 TB Treatment regimens

- Patients diagnosed with active TB either at recruitment or during the study but who are not on IPT will be treated according to national guidelines.
- Patients who develop TB while on IPT will be treated with a retreatment TB regimen.

7 Workforce turnover

The cohort will be an open one, so that members can join as well as leaving during the study. Workforce turnover will be monitored weekly at each site, so that all new employees will be identified promptly

The known workforce at each site will be checked 3 monthly against a new payroll record, to double-check that the project list is up-to-date

7.1 New employees

New employees joining a study company after initiation, but before the study end date will be invited to participate in all of the following:

- Baseline questionnaire and specimens
- VCT
- Preventive therapy if HIV-positive and eligible for isoniazid or cotrimoxazole

Isoniazid can still be commenced even if there is insufficient time to complete for follow-up, provided that the employee is willing to come to BRTI once a month to complete the treatment course

7.2 Employees leaving the study workforce

- The date and reason for leaving will be recorded for subsequent cohort analyses
- the study team will continue supplying isoniazid and following-up employees leaving employment while on isoniazid until the full 6 months of therapy has been completed.
- In such cases the client will be reviewed monthly either at their home address, or at BRTI, until treatment is complete
- a supply of medication and follow-up will be maintained for 3 months for employees who leave while taking cotrimoxazole, and the client will be assisted in securing another source of this medication, to which all symptomatic HIV-positive persons in Zimbabwe are entitled

8 Outcome measures

8.1 Primary outcomes

a) To compare VCT uptake and the proportion of HIV-positive employees in each arm who are identified and screened for preventive therapy eligibility

If the proportion of HIV-positive employees screened for preventive therapy eligibility is significantly different under the two strategies, then comparison of the following between the two arms will also be primary outcomes:-

b) absenteeism rates due to ill-health
c) incidence rates of severe morbidity
d) incidence rates of TB during the last 12 months of the intervention

If significant differences exist in b) ➔ d) then the
e) cost-effectiveness of businesses providing this intervention for their employees will also be investigated

8.2 Secondary outcomes

The difference between the two arms in:-
a) absenteeism rates due to ill-health among all HIV-positive employees
b) incidence of serious morbidity events among all HIV-positive employees
c) prevalence of active TB detected on a cross-sectional survey at the end of the intervention at each site.

8.3 Case definitions

In the case of ill-health, diagnoses will be based on those defined by the syndromic management guidelines. Of note are the following:

8.3.1 TB

Definite:	Culture confirmed as *Mycobacterium tuberculosis* with > 5 colonies, *plus* radiological or clinical evidence of TB disease, *or* persistent TB excretion on two separate occasions
Probable:	Clinically compatible and culture positive (without organism identification) or culture negative / not done and smear positive on 2 or more specimens
Possible:	Meets the above case-definitions for a TB suspect *and* response to TB treatment for an illness that is *either* clinically compatible *and/or* associated with a single positive smear

TB diagnosed at the time of autopsy will be counted as definite TB.

A more stringent definitions of TB disease will be used for the cross-sectional prevalence survey, when confirmation of definite TB will require 2 or more positive sputum cultures taken on separate occasions, and smear positive TB disease will be confirmed with a second set of sputum smears.

8.3.2 Severe morbidity

This is defined as ill-health of sufficient gravity to result in:

- admission to hospital *or*
- death *or*
- medical retirement *or*
- 5 or more sequential working days of sick leave

8.4 Analysis

As the primary outcome of interest is absenteeism, the analysis will need to control for differences by site in the following factors:

- pre-intervention rates of absenteeism due to ill-health
- HIV prevalence

The following are potentially important variables that could explain some of the pre-intervention variability in absenteeism rates:

- male: female ratio (in part by different HIV prevalence rates and in part HIV-independent)
- pre-existing on-site clinic
- private versus para-state organisations
- heavy versus light industry
- routine recording of absenteeism by the hour or by the day
- medical retrenchment policy

Information on all of the above variables will be collected for inclusion in the final analysis.

At the time of recruitment a baseline blood specimen will be obtained from all study participants for anonymous HIV testing. Results will be kept separately and confidentially until the end of the study, when they will be linked with other study data with no loss of anonymity, as described in section 9.1.1.2. All participants will also be encouraged to have VCT.

9 Laboratory methods

9.1 Surveillance specimens

9.1.1 Processing and storage of baseline blood specimens

Blood specimens will be taken into acid citrate dextrose vacutainer tubes that bear the name and company number of the individuals. These will be transported to the laboratory accompanied by the completed BD01 questionnaire, signed consent form and sputum specimen.

A laboratory number and anonymous HIV test number will be allocated and HV01, BD02 and BD03 forms filled in by the laboratory staff. Whole blood specimens will centrifuged in the original vacutainers.

9.1.1.1 Plasma

A sample of plasma will be removed for anonymous HIV testing (see below). 3.6 mls of the remaining plasma will then be aliquoted into two cryotubes and frozen at -20°C, stored under the HV01 laboratory number.

9.1.1.2 Baseline anonymous HIV tests

Once a dedicated HIV test record number has been and forms BD02 and BD03 filled in and separated specimens will be processed and results stored using the fields defined in BD03, which include a dedicated HIV test number that can be traced to an individual through the "linking" form BD02. Forms BD03 and BD02 will be entered into different databases, and physically stored in separate locked cabinets.

Specimens will be tested for antibodies to HIV using Abbott Determine™, a rapid HIV test of high sensitivity and specificity. For quality control purposes, one specimen in 10 will be double-checked using a second rapid HIV test, Trinity Biotech Unigold™.

At the end of the study the database tables containing data from BD02 and BD03 will be imported into the same database and merged with other study data using a computer programme that will strip all personal identifiers immediately after merging the files.

9.1.1.3 Buffy coats

Following the initial centrifugation and removal of plasma, the buffy coat will be taken off by ringing round the centrifuge tube with a plastic transfer pipette, and will then be placed in a centrifuge tube, washed once with PBS and re-centrifuged. After decanting the PBS, the buffy coat will be re-suspended to 4mls in PBS, divided into two cryotubes and frozen at -70°C, stored under the HV01 laboratory number.

9.1.1.4 Baseline sputum specimens

Sputum specimens provided at the one-to-one interview will be allocated a laboratory number, and transferred and heat fixed onto a glass slide using an orange stick to select the most mucoid area. Unlike the diagnostic smears, these specimens will not be decontaminated or concentrated, and the specimen will be discarded once the smear has been made. Smears will then be stored unstained and unexamined in numbered, dedicated, smear boxes separately from other diagnostic smears.

9.1.2 Ongoing routine 2 monthly sputum smears

These will be collected every 2 months from all employees of study sites who are willing to provide specimens

- **Specimens should be collected from *all willing subjects* regardless of whether they have submitted sputum before, or joined in any other aspect of the project or not**
- **It will be the responsibility of the clinic nurse to ensure collection of sputum specimens from as many participants as possible, aiming for >90% participation at each time point**
- **A specimen collection assistant will be available to *assist* with collection and transportation of the specimens**

They will be processed and stored in the same fashion as the baseline sputum specimens (detailed in Section 9.1.1.4 above.

It is important for each of the participants, specimen collectors and laboratory staff to realise that these smears will not be read until the end of the study, even if the specimens appear diseased. The study aims to establish a representative "sputum smear library", and if laboratory staff select slides to read on their own initiative before the final cross-sectional study, then the sample will become unrepresentative. In any case a single smear is sub-optimal for diagnostic purposes: 3 smears should be submitted for diagnosis, and participants may be lulled into a false sense of security if told that their smears will be examined when this is not being carried out systematically.

Instead it should be stressed to all participants that these specimens ***will not*** be used for diagnostic purposes, and that they need to submit 3 more specimens through the clinic nurse if they have suggestive symptoms, such as cough for over 3 weeks or bloody sputum.

9.2 Diagnostic HIV tests

Most diagnostic HIV-testing will be carried out in the industrial clinics. However, the laboratory will also provide some diagnostic services, and will also be responsible for a quality assurance programme:-

9.2.1 VCT in the industrial sites

- Written consent will be required from all clients
- Following brief pre-test counselling, a risk assessment will be carried out and form VT01 filled in
- 3 drops of blood from a finger prick wound or venous blood sample will be taken onto a pre-prepared #3 Whatman Filter Paper to provide a dried blood spot (DBS) record for each client
- a pipette will then be used to obtain 3 more drops of blood for HIV-testing: a second finger prick may be necessary at this stage
- Two rapid HIV tests will then be run simultaneously for each client
  - Abbott Determine™
  - Trinity Biotech Unigold™
- Concordant results (both test kits positive, or both test kits negative) will be communicated to the client without further delay
- Discordant results, or an indeterminate result on one test kit, will be communicated as an uninterpretable result, and will be followed by a request for venous blood to be sent into the central laboratory where the HIV status will be resolved (see below)

9.2.2 Routine quality assurance

One day in 9, according to a pre-defined schedule, all VCT will be carried out using venous blood specimens. An HV01 form will be filled in and sent in with the remaining blood to the laboratory requesting QA.

The laboratory staff will repeat the same HIV testing algorithm. Any discordant results will be communicated immediately, and a fresh specimen of blood requested from the client.

9.2.3 Laboratory diagnostic HIV tests

These will be requested on QA days, and to resolve HIV status in the case of discordant or indeterminate results.

The laboratory will initially repeat the same protocol as used on site and detailed in section 9.2.1. In cases where a clear concordant result is obtained the result will be taken as final with no further tests. Where a discordant or indeterminate result is obtained again, then a "tie-breaker" third rapid test (capillis or dipstick) will be used. If two of the three positive tests will be reported as positive.

9.3 *Diagnostic TB smear and culture methods*

9.3.1 Diagnostic TB specimens

TB suspects will be managed as indicated in IMAI. Patients with a cough > 3 weeks, or other features suggestive of TB (such as fever, or weight loss) will be investigated with 3 sputum specimens for microscopy and culture.

9.3.2 Diagnostic TB cultures

9.3.2.1 Microscopy

Smears will be made from concentrated decontaminated sputum, stained with Auramine-O and examined under fluorescent microscopy by two readers. Positive slides will be confirmed with ZN staining.

Slides will be graded for positivity (scanty, 1+, 2+, 3+, 4+) according to WHO guidelines

9.3.2.2 Primary culture methods

Diagnostic sputum specimens will be decontaminated using the standard 4% NaOH method (see WHO handbook), and concentrated with centrifugation at 3000 rpm for 15 mins.

The pellet will be resuspended and an aliquot inoculated onto Lowenstein-Jensen (LJ) media and into Kirchner's media. The LJ slopes and Kirschner's will be inspected weekly for growth for up to 8 weeks. All positive cultures will be confirmed as mycobacteria using Ziehl-Neelsen staining and then undergo identification of the mycobacterial organism. Primary drug susceptibility testing will not be routinely carried out.

9.3.2.3 Identification methods

Positive slopes will be sub-cultured onto LJ slopes and

a) Sent to Bulawayo laboratory for identification, plus
b) Identified on-site

Colonial and microscopic morphology, and physico-chemical growth differential tests will be used to classify organisms as *M. tuberculosis* complex or mycobacteria other than TB.

9.3.2.4 Storage of original specimens

Decontaminated sputum specimens will be stored at -20°C until positive or negative culture results are confirmed. In the case of contaminated cultures, the decontaminated specimens will be retrieved and recultured after repeat decontamination.

9.3.2.5 Storage of cultures

All positive cultures will be stored at -70°C in glycerol.

10 Treatment interruption

If there are short interruptions in preventive therapy, the patient should be counselled, and the therapy continued so that the patient receives at least 6 months therapy in total, as guided by IH01.

If the patient interrupts for more than a month on two occasions, despite counselling, stop the preventive therapy. WHO recommends that the 6-month course should be completed within a year of its initiation.

Appendix 1. Management of adverse events, drug interactions and contraindications to isoniazid and cotrimoxazole

Isoniazid preventive therapy

Side effects	Treatment modification
• Drug induced hepatitis manifest by jaundice, nausea and vomiting, right-upper quadrant pain, confusion, AST increased 5x greater than upper limit of normal, dark urine	• Stop INH, and do not reintroduce. Warn patient to report that they react to isoniazid should TB treatment be started at any time in the future
• INH induced seizures	• Stop INH
• Peripheral Neuritis	
○ Mild	• Add pyridoxine 25 mg od to INH
○ Severe	• Stop INH and give pyridoxine 25 mg bd for 2 weeks
• Probable drug-induced skin eruptions	• Withdraw all medication and prescribe antihistamines as required.<br>• When rash has subsided, reintroduce medications one at a time at 2-day intervals (leaving the most suspect drug until last)

INH drug interactions

- Antiepileptics (phenytoin, carbamazepine) – INH inhibits the metabolism of carbamazepine and phenytoin and so may potentiate their effect. Also carbamazepine may increase the risk of INH hepatotoxicity.
- Ketoconazole: INH may reduce the plasma ketoconazole levels.
- Any drug which also may cause peripheral neuropathy should be avoided / used with care to avoid potentiation of this risk (e.g. metronidazole, didanosine, thalidomide)

Contra-indications

- Chronic hepatitis
- Severe peripheral neuropathy
- Previous hypersensitivity to INH

Cautions

The risk of hepatotoxicity due to isoniazid is greater in individuals with:-

♦ age over 35 years
♦ high alcohol intake (daily drinking)
♦ chronic liver disease, including hepatitis B

Cotrimoxazole prophylaxis

Side effects	Treatment modification
• Skin hypersensitivity:<br>o Erythema multiforme<br>o Stevens-Johnson Syndrome<br>o Toxic epidermal necrolysis	• Stop treatment<br>• Refer to hospital if reaction severe<br>o high fever,<br>o mucous membrane involvement or skin shedding
• Haemotological toxicity<br>o Anemia,<br>o Neutropenia<br>o Thrombocytopenia	• If neutrophils < 1.0 or platelets < 100<br>o stop treatment<br>o give folic acid 15 mg daily and repeat blood count in 1 week<br>o refer to hospital if no improvement<br>o do not reintroduce cotrimoxazole
• Nausea, vomiting, diarrhoea	• Try giving tablets at night, or alternate days<br>• Check liver function tests for hepatitis if continued or severe<br>• If there is no evidence of hepatitis, try to continue but stop treatment if not tolerated
• Raised Liver enzymes<br>o (greater than 5x normal)	• Stop treatment

Drug interactions
- Anticoagulants: cotrimoxazole enhances the effect of warfarin
- Antidiabetics: cotrimoxazole enhances the effect of sulphonylureas
- Antiepileptics: cotrimoxazole increases plasma levels of phenytoin

Contra-indications
- Pregnancy in the first trimester
- Porphyria
