# Supplementary material for: Uptake of Workplace HIV Counselling and Testing: A Cluster-Randomised Trial in Zimbabwe
Source: PLoS Med. 2006 Jul 4;3(7):e238. doi: 10.1371/journal.pmed.0030238 (PMC1483908; doi:10.1371/journal.pmed.0030238)
Supplement: Supplementary file 2 [file pmed.0030238.sd001.doc]

|  | **Item** | **Descriptor** | **Reported on** |
| --- | --- | --- | --- |
|  | **number** |  | **page number** |
| **Title and abstract** | 1 | How participants were allocated to interventions (eg, "random allocation", "randomised", or "randomly assigned"). *Specifying that allocation was based on clusters* | 1 and 2 |
| **Introduction** | | | |
| Background | 2 | Scientific background and explanation of rationale, *including the rationale for using a cluster design* | 4 |
| **Methods** | | | |
| Participants | 3 | Eligibility criteria for participants *and clusters* and the settings and locations where the data were collected. | 5 |
| Interventions | 4 | Precise details of the interventions intended for each group*, whether they pertain to the individual level, the cluster level or both,* and how and when they were actually administered. | 6 |
| Objectives | 5 | Specific objectives and hypotheses *whether they pertain to the individual level, the cluster level or both,* | 5 |
| Outcomes | 6 | Clearly defined primary and secondary outcome measures whether they pertain to the individual level, the cluster level or both, and, when applicable, any methods used to enhance the quality of measurements (eg, multiple observations, training of assessors, &c). | 5 |
| Sample size | 7 | How *total* sample size was determined *(including method of calculation, number of clusters, cluster size, a coefficient of intracluster correlation, and an indication of its uncertainty* and, when applicable, explanation of any interim analyses and stopping rules. | 5 |
| Randomisation | | | |
| Sequence generation | 8 | Method used to generate the random allocation sequence, including details of any restriction (eg, blocking, stratification*, matching*). | 5 |
| Allocation concealment | 9 | Method used to implement the random allocation sequence, *specifying that allocation was based on clusters rather than individuals* (eg, numbered containers or central telephone), clarifying whether the sequence was concealed until interventions were assigned. | 5 |
| Implementation | 10 | Who generated the allocation sequence, who enrolled participants, and who assigned participants to their groups. | 5 |
| Blinding (masking) | 11 | Whether or not participants, those administering the interventions, and those assessing the outcomes were aware of group assignment. If not, how the success of masking was assessed. | NA |
| Statistical methods | 12 | Statistical methods used to compare groups for primary outcome(s); *indicationg how clustering was taken into account* methods for additional analyses, such as subgroup analyses and adjusted analyses. | 8 |
| **Results** | | | |
| Participant flow | 13 | Flow of *clusters and individuals* through each stage (a diagram is strongly recommended). Specifically, for each group, report the numbers of participants randomly assigned, receiving intended treatment, completing the study protocol, and analysed for the primary outcome. Describe protocol deviations from study as planned, together with reasons. | 24 |
| Recruitment | 14 | Dates defining the periods of recruitment and follow-up. | 6 |
| Baseline data | 15 | Baseline demographic and clinical characteristics of each group *for the individual and cluster level as applicable*. | 20 |
| Numbers analysed | 16 | Number of *clusters* and participants (denominator) in each group included in each analysis and whether the analysis was by "intention to treat". State the results in absolute numbers when feasible (eg, 10/20, not 50%). | 9, 10 ,22, 24 |
| Outcomes and estimation | 17 | For each primary and secondary outcome, a summary of results for each group, and the estimated effect size and its precision (eg, 95% CI). | 9,10,22 |
| Ancillary analyses | 18 | Address multiplicity by reporting any other analyses performed, including subgroup analyses and adjusted analyses, indicating those prespecified and those exploratory. | 10, 11 |
| Adverse events | 19 | All important adverse events or side-effects in each intervention group. | 9 |
| **Discussion** | | | |
| Interpretation | 20 | Interpretation of the results, taking into account study hypotheses, sources of potential bias or imprecision and the dangers associated with multiplicity of analyses and outcomes. | 11, 12,13 |
| Generalisability | 21 | Generalisability (external validity) of the trial findings *to individuals and/or clusters as relevant* | 13, 14 |
| Overall evidence | 22 | General interpretation of the results in the context of current evidence. | 12,13,14 |
| **Checklist of items to include when reporting a randomised trial** | | | |
